# Effects of a 12-week increased hip flexion gait exercise intervention in individuals with obesity and knee osteoarthritis: a randomized crossover feasibility study

**DOI:** 10.3389/fspor.2026.1791400

**Published:** 2026-04-08

**Authors:** Nuno Oliveira, Jon Stavres, Chuang-Yuan Chiu, Austin J. Graybeal

**Affiliations:** 1 School of Kinesiology and Nutrition, University of Southern Mississippi, Hattiesburg, MS, United States; 2 Department of Kinesiology, Nutrition, and Health, Miami University, Oxford, OH, United States; 3 Sports Engineering Research Group, Sheffield Hallam University, Sheffield, United Kingdom; 4 Department of Kinesiology, Harris College of Nursing and Health Sciences, Texas Christian University, Fort Worth TX, United States

**Keywords:** exercise, gait, knee, obesity, osteoarthritis

## Abstract

**Introduction:**

The novel increased hip flexion gait (HFgait) exercise modality involves specific neuromuscular and biomechanical characteristics that might be beneficial for individuals with knee osteoarthritis (OA). The current study examined the efficacy and feasibility of a 12-week HFgait exercise program in patients with obesity and symptomatic knee OA.

**Methods:**

Double-arm crossover design with three timepoint measurements. Twelve participants (Age: 62.9 ± 10.6 yrs, BMI: 31.7 ± 6.0 kg/m^2^; 72% female) with obesity, self-reported clinician diagnoses of knee OA and moderate to severe pain score (≥ 5) from the Western Ontario and McMasters Universities Index (WOMAC) enrolled in a 12-week, supervised HFgait intervention with sessions occurring 3-d/wk. Feasibility, motor function, symptomatic burden, balance, strength, cardiometabolic health, body composition, and cardiovascular fitness were assessed.

**Results:**

6MWT [post-pre intervention difference, mean ± SD: *Δ* 52.2 ± 26.5 m, 95% CI: (33.1, 71.4)], TUG [*Δ* 0.9 ± 1.2 s, (0.03, 1.8)], WOMAC-Pain [*Δ* 3.4 ± 3.7, (0.7, 6.1)], WOMAC-Function [*Δ* 7.6 ± 9.6, (0.6, 14.5)] and WOMAC-Total [*Δ* 11.4 ± 13.5, (1.6, 21.2)] significantly improved with the HFgait intervention. A significant main effect of sequence of control-intervention was observed for WOMAC-Pain [2.0 ± 3.2, (0.1, 3.9)]. Additionally, hip circumference [*Δ* −2.8 ± 3.8 cm, (−4.9, −0.6)], body fat percentage [*Δ* −1.1 ± 1.7%, (−2.2, −0.01)], and lumbar spine bone mineral density [*Δ* 0.04 ± 0.05 g/cm^2^, (0.01, 0.1)] improved with the HFgait intervention.

**Discussion:**

A 12-week HFgait intervention showed high retention and adherence with no adverse events. The HFgait exercise program improved motor function, OA symptoms, and body composition. Trial registration number: clinicaltrials.gov: NCT05997862.

## 1 Introduction

Osteoarthritis is a chronic condition that is characterized by pain, impaired function, and reduced quality of life (1). In the U.S., estimates suggest that almost 32.5-million adults have been clinically diagnosed with osteoarthritis (OA). Previous studies have reported that nearly half of adults with osteoarthritis also have arthritis-attributable severe joint pain (2). In addition to pain, individuals with osteoarthritis experience stiffness and decreased range of motion (ROM) of the joints, leading to a loss of functional independence and reduced mobility (3).

Exercise is a critical element in the treatment and improvement of function, pain and quality of life of individuals with osteoarthritis (4, 5). The American College of Rheumatology, European League Against Rheumatism, American Academy of Orthopedic Surgeons and the Osteoarthritis Research Society International all strongly recommend physical exercise as non-pharmacologic treatment for individuals with osteoarthritis (4, 5). Moreover, a positive relationship has been established between exercise-related health benefits and the intensity of that exercise (6). While self-selected recreational running has been associated with improvements in pain in individuals with osteoarthritis, these typically low-intensity running loads do not meet American College of Sports Medicine guidelines for high-intensity exercise, and consequently, might prevent optimal cardiovascular benefits and body composition or optimal physical function and pain improvements (7, 8).

Due to its time efficiency and flexibility as an exercise modality, high intensity interval training (HIIT) has the potential to address some of the current limitations in meeting high-intensity exercise guidelines and adherence during exercise interventions in individuals with osteoarthritis (9). Golightly et al. (10) recently reported good adherence and tolerability in a feasibility study of a 12-week HIIT program in individuals with knee osteoarthritis. However, it is not only important to consider the symptomatic burden during the exercise, but also the type of exercise modality that might maximize exercise benefits, such as maximizing cardiorespiratory fitness and decreasing OA symptomology. For example, in Golightly et al. (10) study, only 3 out of 24 participants (14%) chose treadmill running to cycling during the HIIT program. This indicates a clear preference towards cycling when running is the alternative in individuals with OA, likely due to the weight bearing component of the task associated with high-intensity running (11). Despite allowing for persons with OA to exercise at high intensities, interventions employing cycling as the exercise mode have reported a lack of effectiveness for improving physical function, daily activity, quality of life, and mobility in individuals with knee osteoarthritis (12). Biomechanical and neuromuscular characteristics of the stationary cycling movement have been suggested as possible explanations and include: 1) limited improvement of knee joint stiffness due to limited knee ROM during cycling; 2) unobserved improvements in a number of components crucial for global motor function (e.g., neuromuscular control) from the lack of collaborative whole-body work; and 3) restricted movement variability that effectively limits improvement in balance and coordination due to the closed chain nature of stationary cycling that elicits lower-limb movement constraints.

Increased Hip Flexion Gait (HFgait) has the potential to be a viable alternative to running and stationary cycling. HFgait addresses the weight bearing limitations of traditional running, and the biomechanical and neuromuscular limitations of cycling. During HFgait, individuals walk on a treadmill and increase intensity (metabolic cost) by increasing hip flexion while walking (13, 14). This removes the flight phase associated with running and reduces knee joint contact forces (15). The resulting activity is an open chain exercise with several degrees of freedom that involves whole-body movement (upper limbs required to maintain balance) and coordination between body segments. In particular, HFgait involves larger hip and knee sagittal plane ROM than cycling, and the limited ROM during cycling has been associated with increased severity of disability in patients with hip and knee OA (16, 17). Although the metabolic costs and biomechanics of HFgait have been studied (13–16), the feasibility and the effects of this type of exercise in individuals with knee OA has not yet been investigated. Determining the feasibility and preliminary efficacy of this type of protocol will inform the design of larger randomized controlled studies and hypothesis generation. Therefore, the purpose of this study was to 1) examine the feasibility and adherence of a 12-week HFgait HIIT program in patients with obesity and symptomatic knee OA; and 2) evaluate the changes in motor function, symptomatic burden, balance, strength, cardiometabolic health, body composition, and cardiovascular fitness resulting from the 12-week HFgait HIIT program.

## 2 Methods

### 2.1 Participants

Participants were prospectively recruited for this randomized, feasibility study using word of mouth (community, rehabilitation clinics, University, and churches) and contact with previous participants. Participants received monetary compensation for their participation in the study. Inclusion criteria for participation included being 30–75 years of age; a body mass index (BMI) > 30 kg/m^2^; waist circumference (WC) > 102 cm (men)/ > 88 cm (women) (18); or % body fat (%fat) > 25 (men)/ > 34 (women) (19), moderate to severe pain (pain score ≥5) on the Western Ontario and McMasters Universities Index (WOMAC), and medical clearance if self-reported history of cardiovascular events or heart disease. Participants were excluded if they had rheumatoid arthritis, fibromyalgia, gout, diabetes, untreated hypertension or a total knee replacement; and if they had been hospitalized for a cardiovascular event in the previous 6-months, received knee injections in the previous 3-weeks; or self-reported participating in greater than 150-min of moderate exercise per week.

A total of 22 individuals were screened via phone for eligibility. Of those participants, 14 reported being diagnosed with knee OA by a physician and were enrolled in the study. Of the 14 participants, 12 individuals completed the study in its entirety (*N* = 1 discontinued due to inability to safely walk on the treadmill; *N* = 1 discontinued for undisclosed personal reasons) (Table 1). Prior to testing, all participants signed a written informed consent approved by the University's Institutional Review Board. This study was registered at clinicaltrials.gov (NCT05997862).

**Table 1:** Participant characteristics (mean ± SD).

Participant Characteristics	Total *n* = 12
Age (yrs)	62.9 ± 10.6
Height (cm)	169.7 ± 5.6
Weight (kg)	91.1 ± 16.5
BMI (kg/m^2^)	31.7 ± 6.0
Sex	
Male	4
Female	8
Ethnic Group	
Caucasian	11
Black	1

### 2.2 Experimental design

This study used a randomized crossover design, consisting of two arms: Intervention and Control. The crossover comprised a twelve-week HFgait intervention period and a 12-week no-exercise control period. Participants were randomly allocated to one of two possible sequences (Intervention→Control or Control→Intervention). Therefore, each participant completed three testing sessions: one control, one pre-intervention, and one post intervention. Participants assigned to the Intervention→Control sequence first completed the pre-intervention testing visit, followed by the 12-week HFgait program and subsequent post-intervention session. They then completed a 12-week control period with no intervention, after which follow-up testing was conducted for the control session. Participants assigned to the Control→Intervention sequence completed the control session, then returned after 12-weeks of no intervention to complete their pre-intervention testing visit. Participants then completed the 12-week HFgait program and subsequent post-intervention testing. This type of study design allows for a shorter study enrollment that can both capture intervention effects while removing possible carryover. At each testing session, participants were assessed for symptomatic burden (WOMAC pain, WOMAC stiffness, and WOMAC function), physical function [6 min walking test [6MWT], Timed-up-Go test [TUG]], strength, balance [single leg stance and center of pressure (CoP) analysis], walking economy, peak oxygen consumption (VO_2peak_), cardiometabolic health, and body composition. Body composition and cardiometabolic assessments also followed abstention from food (8-h) and planned exercise (12-h).

### 2.3 Intervention

All training was performed on a treadmill (TR5000, LifespanFitness, Salt Lake City, UT) under one-on-one supervision with trained research staff. Participants trained three times a week for twelve weeks for a total of 36 training sessions. Training sessions took place a minimum of 24-hours apart, preferentially scheduling visits on non-consecutive days each week. Training intensity was set at 80% heart rate reserved (HRR) using age-predicted maximal HR. Resting heart rate was measured every week after 5 min of seated rest. At the beginning of each exercise session, participants performed a dynamic calibration that determined the participant's maximum hip flexion angle during treadmill walking. This step determined the hip flexion target that participants had to meet during the session (14). Participants were instructed to ‘bring their knees up as much as possible without feeling pain’ while walking on the treadmill. After calibration, the exercise session consisted of ten repetitions of 1-min bouts at 80% HRR with 1-min rest periods between each work interval. Participants were also instructed to keep outside activity and diet consistent with what they were doing prior to study enrollment.

### 2.4 OA symptomatic burden

Participants completed the WOMAC questionnaire at each testing point. The WOMAC is a 24 item questionnaire that has been shown to be valid and reliable in individuals with knee OA (20). It includes pain (5 items), stiffness (2 items), and function (17 items) subscales, which were rated on a 5-point Likert scale from no symptoms (0) to extreme symptoms (4).

### 2.5 Physical function

The 6MWT and TUG tests were used to assess participants’ physical function. The 6MWT is a walking test where participants are asked to “walk as far as you can during 6-minutes”. It is recommended by the Osteoarthritis Research Society International to assess physical function in patients with knee OA as a test of submaximal aerobic capacity and ability to walk over long distances (21) showing good psychometric properties (22, 23). The Timed Up and Go (TUG) test is a simple and quick test to assess patients’ functional mobility. The Osteoarthritis Research Society International (OARSI) includes the TUG test in a set of five performance based tests of physical function for individuals diagnosed with hip or knee OA (21). This test has demonstrated good measurement properties in individuals with OA (24).

### 2.6 Strength

Knee extensor and flexion strength have been associated with an increased risk of symptomatic and functional deterioration and of worsening tibiofemoral OA (25). Maximal isometric knee extensor and flexor strength were assessed using an isokinetic dynamometer (Biodex Medical Systems, Inc, Shirley, NY) at 60° knee flexion. Participants performed three 5-seconds of maximal extension and flexion trials with a 30-second rest period between trials. Participants received strong verbal encouragement to ‘push as hard as you can’ during trials. Maximum extension and flexion across the three trials were recorded.

### 2.7 Balance

For the single leg stance (SLS), participants maintain unilateral stance on one limb without touching the free limb to the ground or performing excessive trunk or upper body movements for a maximum duration of 30 s. Tasks with a single leg stance have been used in knee OA cohorts in previous studies (26). Three trials with eyes open and eyes closed for each leg were performed. The maximum time across the three trials for each condition was recorded.

Center of Pressure (CoP) measurements have been previously used as an important measure of postural balance in individuals with knee OA (27). In particular, ellipsoid area (ELL Area) and path length (PL) have been positively correlated with OA severity (27) and degenerative changes in OA (28). For each trial, the anteroposterior and mediolateral coordinates of the COP were derived from the force and moment measured by a force platform (AMTI, Watertown, MA, USA). Center of pressure (CoP) displacement during bipedal stance were measured under two conditions: 1) eyes open (participants were asked to look at a target 2.5 m in front of them); and 2) eyes closed.

### 2.8 Anthropometrics and body composition

Height and weight were measured using a calibrated, digital stadiometer (seca 286, seca, Hamburg Germany). WC and hip circumference (HC) were measured at the level of the iliac crest and widest lateral portion of the hips, respectively, by the same trained investigators using a flexible aluminum tape measure (29).

Total and regional body composition estimates including %fat, fat mass (FM), fat-free mass (FFM), lean soft tissue (LST), visceral (VAT) and subcutaneous fat (SAT) area, and bone mineral content (BMC) and density (BMD) were obtained using whole-body DXA scans (GE Lunar iDXA, GE Medical Systems Ultrasound & Primary Care Diagnostics, Madison, WI) with version 18 enCORE software. All scans were performed in accordance with established guidelines (30), including daily calibration using phantom scanning procedures. Reflection scanning procedures were used for larger participants unable to fit within the DXA's lateral scanning dimensions, which has shown to produce minimal error (31, 32).

### 2.9 Cardiometabolic health parameters

Systolic and diastolic blood pressure, as well as resting HR, were collected after ≥5-min of seated rest using a digital blood pressure monitor. Then, ∼40-*μ*l of capillary blood were collected from the participant's finger and placed into a single-use cassette, which was then inserted into a validated, point-of-care capillary blood analyzer (Cholestech LDX, Abbot, Abbott Park, IL) to produce measurements of fasting high-density lipoprotein cholesterol (HDL-C), low-density lipoprotein cholesterol (LDL-C), triglycerides (TRG), and blood-glucose (FBG). An additional ∼5-μl of capillary blood were collected into a separate capillary pipette and placed into a single-use cassette for measurements of glycated hemoglobin (HbA1C) using another validated analyzer (A1CNow+; pts diagnostics, Whitestown, IN).

### 2.10 Cardiorespiratory fitness

Cardiorespiratory fitness was quantified as peak oxygen consumption (VO_2peak_) and walking economy (kcal/mile). VO_2peak_ was evaluated using a Balke treadmill exercise testing protocol (33). This protocol maintains a constant speed during the test and progressively increases treadmill inclination (grade) until exhaustion. Respiratory gases were monitored breath-by-breath with open circuit spirometry using a calibrated metabolic cart (True One 2400®, Parvo-Medics, Inc., Provo, UT). Heart rate (HR) was monitored throughout the duration of the test via a polar HR monitor (Polar Verity Sense, Polar USA, Port Washington, NY, USA). For participants with a history of cardiovascular events or heart disease (*n* = 3), the VO_2peak_ test stopped at 75% of age-predicted maximal HR. This cutoff value was selected in a conservative approach to maintain participant safety and limit unnecessary cardiovascular event risk. For these participants, VO_2peak_ was predicted by linear regression using the collected VO_2_ and HR values. Prior to the start of the Balke protocol, participants walked on the treadmill for 4 min at a self-selected pace. Walking economy was calculated from the average VO_2_ during the final three minutes of the walking phase using the formula kcal/mi*n* = (VO_2_*3.5/1000*5)*(60/mph). Due to equipment malfunction, data from one testing session was lost. Therefore, we only include 11 participants in the VO_2peak_ and walking efficiency analysis.

### 2.11 Statistical analysis

Data were descriptively summarized with mean and standard deviations at baseline, and pre and post intervention timepoints. Linear mixed model analyses were performed to test for main effects of time (baseline, and pre and post intervention timepoints) and sequence (baseline before or after the intervention) for each variable using Sequence as a covariate. When a significant main effect of Time was observed, Bonferroni *post-hoc* tests were used to test for pairwise comparisons. Cohen's *d* effect sizes between pre and post intervention were calculated. Small, moderate and large effect sizes were taken as 0.2, 0.5 and 0.8 respectively. All data were analyzed using jamovi (2.7.16), an open-source statistical software (The jamovi project, 2026, https://www.jamovi.org), or R Studio version 2026.1.0. The criterion for statistical significance for all tests was set *a priori* at *α* = 0.05.

## 3 Results

### 3.1 Feasibility

Two participants withdrew from the HFgait intervention: one participant revealed a severe inability to walk safely on the treadmill; one participant completed 6 weeks but discontinued participation due to personal reasons. All other participants that completed pre-post intervention and control testing completed all 36 visits. No adverse events related to the HFgait intervention or HFgait exercise were reported.

### 3.2 Effects of HFgait intervention

A significant main effect of time was observed for the 6MWT (*p* < 0.001), TUG (*p* = 0.050), WOMAC-Pain (*p* = 0.010), WOMAC-Function (*p* = 0.025), and WOMAC-Total (*p* = 0.019). For 6MWT, *post hoc* Bonferroni comparisons indicated differences between baseline and pre-intervention (*p* = 0.004, d = 0.65), baseline and post-intervention (*p* = 0.014, d = 0.56), and pre-intervention and post-intervention (*p* < 0.001, d = 1.15). For TUG, *post hoc* Bonferroni comparisons indicated differences between pre-intervention and post-intervention (*p* = 0.050, d = 0.80). For WOMAC-Pain, *post hoc* Bonferroni comparisons indicated differences between pre-intervention and post-intervention (*p* = 0.010, d = 1.26). For WOMAC-Function, *post hoc* Bonferroni comparisons indicated differences between pre-intervention and post-intervention (*p* = 0.036, d = 0.93). For WOMAC-Total, *post hoc* Bonferroni comparisons indicated differences between pre-intervention and post-intervention (*p* = 0.024, d = 1.00) (Table 2). A significant main effect of time was observed for HC (*p* = 0.031), %fat (*p* = 0.021), Spine BMD (*p* = 0.023) (Table 3), and diastolic blood pressure (*p* = 0.039) (Table 4). *post-hoc* test revealed that HC (*p* = 0.043, d = −0.97) and %fat (*p* = 0.019, d = −0.67) were significantly lower, while Spine BMD was significantly higher (*p* = 0.031, d = 0.87), post compared to pre-intervention. *post-hoc* tests for diastolic blood pressure were not statistically significant (all *p* ≥ 0.052). No other significant main effects of time or sequence were observed (all *p* > 0.05).

**Table 2:** Functional, balance, strength, cardiorespiratory and body composition variables (mean ± SD) for control and pre-post intervention measurements, and respective *p* values for time and sequence main effects.

Variable	Control	12-week HFgait intervention		Main Effects	
		Pre	Post	Time	Sequence
6MWT (m)	558 ± 41.6	530 ± 45	582 ± 39	**<0.001**	0.821
TUG (s)	6.8 ± 1.7	7.4 ± 1.2	6.4 ± 1.2	**0**.**050**	0.119
Single Leg Stance					
Right EO (s)	21.3 ± 9.5	21.0 ± 10.2	24.8 ± 8.1	0.225	0.414
Left EO (s)	22.1 ± 10.7	19.7 ± 10.6	23.1 ± 8.8	0.189	0.595
Right EC (s)	13.3 ± 10.7	11.3 ± 9.9	12.0 ± 9.8	0.372	0.347
Left EC (s)	8.5 ± 6.9	8.8 ± 8.3	11.9 ± 8.3	0.116	0.655
Strength					
RL Ext. (*N*/m)	142.1 ± 42.6	141 ± 39.2	146 ± 34.6	0.817	0.675
RL Flx. (*N*/m)	73.9 ± 18.3	76.5 ± 19.9	79.8 ± 18.4	0.143	0.208
LL Ext. (*N*/m)	119.8 ± 39.1	134 ± 36.8	139 ± 37.1	0.070	0.530
LL Flx. (*N*/m)	68.7 ± 18.4	70.0 ± 27.0	71.9 ± 17.6	0.598	0.363
Balance (CoP)					
PL EO (cm)	12.1 ± 4.5	11.5 ± 3.3	13.9 ± 7.9	0.323	0.407
AE EO (cm^2^)	0.80 ± 0.58	0.65 ± 0.5	0.65 ± 0.54	0.548	0.637
PL EC (cm)	15.9 ± 9.0	16.4 ± 7.5	17.0 ± 8.4	0.784	0.238
AE EC (cm^2^)	0.74 ± 1.0	1.1 ± 0.86	0.9 ± 0.73	0.250	0.326
WOMAC					
Pain (0–20)	3.8 ± 3.0	6.3 ± 3.1	2.8 ± 2.3	**0**.**010**	**0**.**037**
Stiffness (0–8)	2.3 ± 1.5	2.5 ± 1.4	2.1 ± 1.4	0.648	0.078
Function (0–68)	9.7 ± 7.2	16.2 ± 9.0	8.6 ± 7.2	**0**.**025**	0.081
Total (0–96)	15.8 ± 8.3	24.9 ± 12.3	13.5 ± 10.3	**0**.**019**	0.057
Cardiorespiratory Fitness					
Vo_2peak_ (mL/Kg/min)	28.4 ± 7.6	24.9 ± 9.0	29.8 ± 11.3	0.100	0.965
Walk Economy (Kcal/mile)	109.8 ± 21.2	96.8 ± 33.3	103.3 ± 28.2	0.477	0.116

6MWT, six minute walking test; TUG, timed up and go test; EO, eyes open; EC, eyes closed; RL, right leg; LL, left leg; CoP, center of pressure; PL, path length; AE, area of the ellipsoid; WOMAC, Western Ontario and McMasters Universities Index. Bold indicates statistical significance (*p* ≤ 0.05).

**Table 3:** Body composition variables (mean ± SD) for control and pre-post intervention measurements, and respective *p* values for time and sequence main effects.

Variable	Control	12-week HFgait intervention		Main Effects	
		Pre	Post	Time	Sequence
Weight (kg)	89.0 ± 12.4	88.6 ± 12.9	88.1 ± 12.3	0.624	0.826
BMI (kg/m^2^)	30.8 ± 3.5	30.6 ± 3.9	30.4 ± 3.8	0.535	0.666
Waist (cm)^a^	103.7 ± 8.8	105.9 ± 8.5	105.2 ± 10.9	0.799	0.991
Hip (cm)^a^	112.0 ± 8.7	114.6 ± 9.7	111.8 ± 9.1	**0**.**031**	0.661
Waist-to-Hip ratio^a^	0.93 ± 0.08	0.93 ± 0.05	0.94 ± 0.06	0.616	0.595
Waist-to-Height ratio^a^	0.61 ± 0.05	0.63 ± 0.05	0.62 ± 0.07	0.823	0.638
Body Fat (%)	40.8 ± 7.8	41.2 ± 7.6	40.0 ± 8.1	**0**.**021**	0.580
Fat mass (kg)	35.8 ± 8.5	36.4 ± 9.1	34.6 ± 8.7	0.138	0.769
Fat-free mass (kg)	51.7 ± 9.4	51.4 ± 8.4	51.3 ± 8.4	0.257	0.469
Lean soft tissue (kg)	48.9 ± 9.1	48.6 ± 8.1	48.5 ± 8.2	0.288	0.469
Bone mineral content (g)	2,823 ± 438	2,785 ± 468	2,775 ± 438	0.110	0.640
VAT (g/cm^2^)	189 ± 90	173 ± 101	157 ± 65	0.388	0.220
SAT (g/cm^2^)	242 ± 90	269 ± 132	249 ± 100	0.156	0.816
Trunk %fat	45.0 ± 6.9	45.4 ± 7.3	43.8 ± 7.7	0.072	0.241
Trunk FM (kg)	8.8 ± 2.0	9.1 ± 2.5	8.5 ± 2.1	0.057	0.397
Trunk FFM (kg)	11.1 ± 1.7	11.2 ± 1.4	11.2 ± 1.5	0.790	0.401
Trunk LST (kg)	10.3 ± 1.6	10.4 ± 1.4	10.3 ± 1.4	0.785	0.433
Trunk BMC (g)	858 ± 203	847 ± 217	866 ± 229	0.392	0.461
Android %fat	48.8 ± 6.8	48.9 ± 7.3	47.3 ± 7.7	0.200	0.240
Android FM (kg)	3.4 ± 0.9	3.6 ± 1.1	3.2 ± 0.8	0.091	0.387
Android LST (kg)	3.5 ± 0.7	3.6 ± 0.7	3.4 ± 0.5	0.751	0.816
Gynoid %fat	41.8 ± 10.2	42.4 ± 9.5	41.2 ± 9.9	0.281	0.836
Gynoid FM (kg)	6.0 ± 1.8	5.9 ± 1.7	5.8 ± 1.7	0.464	0.843
Gynoid LST (kg)	7.9 ± 1.4	7.7 ± 1.4	7.8 ± 1.4	0.396	0.284
Android-Gynoid ratio %fat	1.22 ± 0.25	1.18 ± 0.19	1.18 ± 0.21	0.607	0.339
Android-Gynoid ratio FM	0.61 ± 0.19	0.62 ± 0.16	0.58 ± 0.16	0.546	0.165
Appendicular FFM (kg)	24.4 ± 5.7	23.8 ± 5.7	23.9 ± 5.1	0.186	0.482
Appendicular LST (kg)	23.0 ± 5.5	22.5 ± 5.4	22.5 ± 5.0	0.182	0.474
Appendicular BMC (g)	1,387 ± 270	1,391 ± 293	1,356 ± 228	0.961	0.765
Arms FFM (kg)	6.0 ± 2.1	5.9 ± 2.0	5.8 ± 2.0	0.827	0.835
Arms LST (kg)	5.6 ± 2.0	5.6 ± 1.9	5.4 ± 1.9	0.813	0.835
Arms BMC (g)	355 ± 92	355 ± 98	350 ± 79	0.922	0.871
Legs FFM (kg)	18.4 ± 3.7	17.9 ± 3.8	18.1 ± 3.4	0.273	0.338
Legs LST (kg)	17.4 ± 3.6	16.9 ± 3.7	17.1 ± 3.3	0.254	0.327
Legs BMC (g)	1,032 ± 186	1,036 ± 205	1,006 ± 166	0.923	0.726
Bone mineral Density					
Arms (g/cm^2^)	0.89 ± 0.15	0.91 ± 0.12	0.89 ± 0.16	0.431	0.481
Legs (g/cm^2^)	1.30 ± 0.18	1.30 ± 0.17	1.27 ± 0.15	0.850	0.538
Trunk (g/cm^2^)	1.08 ± 0.15	1.07 ± 0.16	1.08 ± 0.15	0.208	0.805
Spine (g/cm^2^)	1.26 ± 0.14	1.23 ± 0.15	1.24 ± 0.13	**0**.**023**	0.582
Pelvis (g/cm^2^)	1.05 ± 0.16	1.05 ± 0.17	1.06 ± 0.16	0.835	0.576
Total (g/cm^2^)	1.27 ± 0.14	1.27 ± 1.40	1.26 ± 0.14	0.537	0.480
Age-Matched Percentile	80.8 ± 20.6	81.3 ± 22.3	80.4 ± 23.9	0.815	0.062
Age-Matched Z-score	1.15 ± 0.91	1.20 ± 0.95	1.18 ± 0.99	0.433	0.302

Bold indicates statistical significance (*p* ≤ 0.05).

^a^ 5 participants missing ≥1 measurements due to technical issues with the measurement modalities.

**Table 4:** Cardiometabolic health variables (mean ± SD) for control and pre-post intervention measurements, and respective *p* values for time and sequence main effects.

Variable	Control	12-week HFgait intervention		Main Effects	
		Pre	Post	Time	Sequence
Systolic Blood Pressure (mmHg)^a^	126 ± 21	126 ± 19	128 ± 15	0.747	0.638
Diastolic Blood Pressure (mmHg)^a^	77 ± 12	84 ± 12	83 ± 10	**0**.**039**	0.277
Resting Heart Rate (bpm)^c^	67 ± 13	72 ± 9	72 ± 14	0.446	0.725
Total Cholesterol (mg/dL)^a^	189 ± 45	178 ± 40	185 ± 37	0.144	0.610
HDL-C (mg/dL)^a^	52 ± 19	49 ± 14	51 ± 13	0.677	0.416
Triglycerides (mg/dL)^a^	116 ± 46	102 ± 47	118 ± 52	0.231	0.498
LDL-C (mg/dL)^a^	114 ± 30	104 ± 29	110 ± 26	0.232	0.732
Non-HDL-C (mg/dL)^a^	137 ± 30	129 ± 32	134 ± 31	0.117	0.787
LDL/HDL ratio^a^	2.27 ± 0.43	2.16 ± 0.59	2.26 ± 0.69	0.744	0.849
FBG (mg/dL)^a^	100 ± 12	96 ± 11	98 ± 9	0.177	0.863
HbA1C (%)^b^	5.2 ± 0.4	5.0 ± 0.3	5.0 ± 0.6	0.471	0.779
10-y CHD Risk (%)^a^	10.9 ± 12.2	12.2 ± 11.9	11.0 ± 13.3	0.486	0.238

Bold indicates statistical significance (*p* ≤ 0.05).

^a^ 5 participants missing ≥1 measurements due to technical issues with the measurement modalities.

^b^ 6 participants missing ≥1 measurements due to technical issues with the measurement modalities.

^c^ all participants missing ≥1 measurements due to technical issues with the measurement modalities.

### 3.3 Carryover effects

A significant main effect of Sequence was observed for the WOMAC-Pain (*p* = 0.037). No significant effects of sequence were observed for any other variable.

## 4 Discussion

The current study introduces a HFgait exercise program for individuals with obesity and knee OA, which consists of a novel exercise modality that increases exercise intensity by increasing maximum hip flexion while walking at a comfortable speed on a treadmill. While HFgait presents theoretical biomechanical and neuromuscular benefits compared to currently available exercise options, the potential for this modality to elicit these benefits has not yet been investigated. Thus, this is the first study to examine the feasibility and effects of a HFgait exercise program on motor function, symptomatic burden, balance, strength, cardiometabolic health, body composition, and cardiovascular fitness.

Recruitment and enrollment were more difficult than initially anticipated. The most common reservation involved the time burden associated with participation in the study (12-weeks, 3 sessions/week). Although, in some cases, this concern was mitigated by the short duration of each exercise session (total duration ≈ 25-minutes; exercising duratio*n* = 10 min). Adherence was perfect for the participants that completed the study. An important aspect that contributed to our high compliance was the flexibility in scheduling exercise sessions. In our program, we were able to schedule exercise sessions according to the participants’ day and time preferences, so long as an interval of 24 h between sessions took place. Additionally, no adverse events were reported during the intervention. During the first 2-weeks of the intervention, we prioritized familiarization with the movements and the structure of the exercise session. During this period, participants were given increased tolerance in meeting their target heart rates (80% HRR) by allowing slower treadmill speeds and giving more leniency in meeting hip flexion targets. As participants became more familiar with the HFgait protocol, treadmill speed and hip flexion targets were manipulated to meet specific target HR at each session.

The main results from the current study were the significant improvements in 6MWT and TUG performance, symptomatic burden, and body composition. During the intervention, participants improved 10 ± 6.5% (52.2 ± 26.3 m) in the 6MWT. This improvement was above the minimal clinical important difference (MCID) for change in 6MWT distance of adults with pathology (14–30.5 m) (34), and just below the minimal detectable change in individuals with OA awaiting arthroplasty (90% CI: 66 m) (23). Improvements in self-selected gait speed have been associated with a reduction in mortality (35). Specifically, Rasmussen et al. (36) reported that reduced gait speed in healthy individuals as early as in their 40s represent comparatively worse physical and cognitive function and an increased rate of physical aging compared with their normal gait speed counterparts. Such evidence has suggested gait speed as a useful ‘vital sign’ for older adults (37). Individuals with symptomatic knee OA consistently report slower gait speeds (38) and are at a high risk of rapid declines in gait speed (39). This is further associated with declines in vigorous physical activity and failure to meet the recommended levels of moderate intensity physical activity (40). This is particularly important because physical activity level has a significant positive relationship with functional performance in adults with knee OA (41). The improvements observed from the HFgait exercise on gait speed might be associated with specific characteristics of the movement. HFgait exercise is mainly driven by increased work at the hip joint, particularly flexion (16), which has been a kinematic parameter associated with increased gait speed (42). Since HFgait involves treadmill walking at comfortable speeds, this might be particularly relevant for specific clinical populations that wish to improve gait speed but have difficulty exercising by walking at increased gait speeds.

Participants also improved in the TUG test. A 11.5 ± 17.8% (0.93 ± 1.3 s) reduction in TUG test scores was observed across participants. This value is slightly lower than previously reported TUG MCID in individuals with knee OA (1.1 s) (43) or individuals with OA awaiting arthroplasty (36.7%) (23), but was greater than the change reported from a 12-week HIIT exercise program (cycling or treadmill) in individuals with knee OA [0.58 s, 95% CI (0.22–0.95)] (10). The TUG is a test recommended by the Osteoarthritis Research Society International (OARSI) to test physical function (44) since it involves several components that are important for functional mobility. It includes transfer tasks, walking, and turning, that require neuromuscular parameters such as power, agility, and balance (45). Additionally, TUG performance has been suggested to be an important predictor of mobility loss and fall frequency in elderly populations with OA (46). Therefore, HFgait might also involve some of the neuromuscular parameters that are associated with TUG performance, which further supports the mobility and functional benefits of this type of exercise.

Significant differences were observed for symptomatic burden. WOMAC-Pain, WOMAC-Function and WOMAC-Total scores improved 3.4 ± 3.7, 7.5 ± 9.9, and 11.4 ± 13.8, respectively. These score improvements are within previously reported MCID in clinical trials involving participants with OA (47–49) and align with previous HIIT interventions that used cycling (10, 50) as the primary exercise modality. Similar to the current study, Smith-Ryan et al. (50) observed significant improvements for the pain and function subscales, but not for the stiffness subscale. It is unclear whether the lack of improvement in stiffness with this type of intervention is due to the characteristics of HIIT or is associated with the low test-retest reliability of the WOMAC stiffness subscale (reliability coefficients, 0.48-0.61) (51). Improvements in WOMAC-Total were likely driven by the changes in WOMAC-Pain and WOMAC-Function subscales. The sequence of control-intervention measurements was significant for WOMAC-Pain. Participants that did Intervention→Control (3.6 ± 3.0) reported lower WOMAC-Pain scores compared with participants that did the control period first (5.7 ± 2.8). This might suggest that there may be some pain improvement from the HFgait intervention that is retained for at least 12-weeks. However, it should be noted that this potential retention effect is inferred from a small sample size using a study design without a dedicated follow-up period for all participants.

Importantly, our concurrent reductions in %fat and increases in lumbar spine BMD represent clinically meaningful adaptations in this cohort with both knee OA and obesity. Excess adiposity is a well-established contributor to OA symptom severity and progression, while the lumbar spine is among the most common sites of age-related bone loss and osteoporotic fracture. The improvement in spine BMD is particularly noteworthy, as HFgait provides repeated axial loading and vertical ground-reaction forces that are mostly absent in non-weight-bearing modalities such as cycling, suggesting that this adapted gait-based intervention may provide musculoskeletal benefits beyond the knee joint alone.

There are a few limitations within the present study that warrant consideration. 1) While the two-arm crossover design used in this study allowed us to assess preliminary effects of HFgait on knee OA, this design did not allow us to include a true washout period between conditions, which affects our ability to interpret our findings. Particularly, retention effects that rely on sequence of treatments. Nevertheless, this is consistent with prior rigorous, longitudinal intervention studies in which the control condition reflects participants’ habitual routines. Such designs are commonly employed because a) they reduce overall study burden and visit frequency, thereby improving retention, and b) any residual effects of one condition are effectively washed out by initiation of the alternate condition and not assessed for ≥12-weeks. 2) This study used a relatively small homogeneous sample size that limits the generalizability of the findings. Additionally, we were not able to collect data from all participants for some of the body composition and cardiometabolic variables which further limits our ability to interpret those outcomes. 3) The current study did not use radiographical evidence for knee OA diagnosis. Therefore, it was not possible to determine the presence of radiographic knee OA or define disease severity based on criteria such as Kellgren-Lawrence. However, we believe the combination of a patient self-reported diagnosis by a physician and current knee pain above our study threshold is a reasonable strategy for identifying patients with knee OA. 4) Adherence to an exercise program may be higher in a study intervention setting than in daily life context.

In conclusion, HFgait seems to be a promising exercise strategy to improve function and knee OA symptoms. In particular, the 12-week HIIT HFgait program resulted in significant improvements in motor function such as walking speed and transfer tasks, and body composition (% body fat, spine density, HC). Improvements in knee OA symptoms included self-reported pain and function. Sequence effects suggest potential retention of pain improvements for as long as 12 weeks after the intervention. Although these effects should be interpreted with caution due to limitations in our study design. Future research should focus on generalizability and confirmation of our preliminary findings by using randomized-controlled trials with a larger sample, additional study arms (e.g., standard-of-care), and a dedicated follow-up period. Optimal deliveries and dosages of HFgait in randomized clinical trials that compare HFgait with standard of care exercise strategies in OA (e.g., walking, cycling, physical therapy) and no-exercise controls should also be investigated.

## Data Availability

The raw data supporting the conclusions of this article will be made available by the authors, without undue reservation.
